# Supplementary figures and images for: A sustainable approach for smallholder farmers: evaluation of plant by-products and industrial waste in wheat cultivation
Source: PeerJ. 2025 Jul 29;13:e19775. doi: 10.7717/peerj.19775 (PMC12315826; doi:10.7717/peerj.19775)

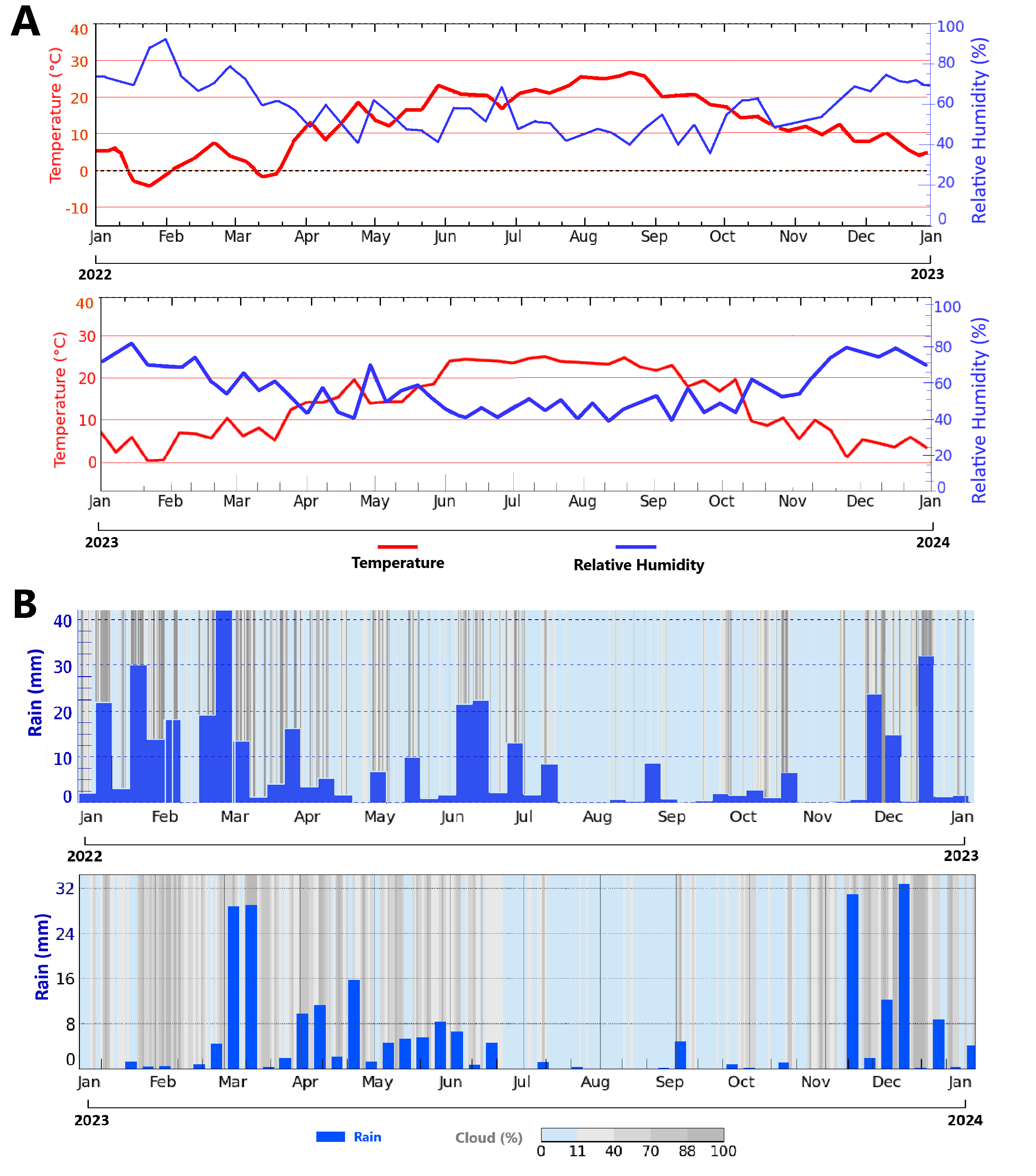

Supplement: Supplemental Information 1 [file peerj-13-19775-s001.png]
